# Associations Between Blood Hemoglobin Concentrations and Cardiometabolic Risk in Middle-Aged Women

**DOI:** 10.1089/whr.2024.0140

**Published:** 2025-01-15

**Authors:** Tazuko Tokugawa, Akihiro Sawada, Satoshi Higasa, Ichiro Wakabayashi

**Affiliations:** ^1^ Department of Respiratory Medicine and Hematology, School of Medicine, Hyogo Medical University, Nishinomiya, Japan.; ^2^ Department of Preventive Medicine, School of Medicine, Hyogo Medical University, Nishinomiya, Japan.

**Keywords:** cardiovascular disease, hemoglobin, metabolic syndrome, polycythemia, women

## Abstract

**Objective:**

Patients with polycythemia have a high risk of thrombo-atherosclerotic diseases. However, it remains to be clarified whether a high blood hemoglobin level is related to cardiometabolic risk in women.

**Methods:**

The overall subjects were 18,410 middle-aged women who had received health checkup examinations at their workplaces. The subjects were divided into four groups of quartiles for hemoglobin levels. Cardiometabolic risk factors were compared in the four quartile groups. Individuals showing abnormally low hemoglobin levels (less than 11.0 g/dL) and/or having a history of therapy for anemia (*n* = 3,690) were excluded from the study.

**Results:**

The prevalence of polycythemia (hemoglobin: higher than 16.0 g/dL) was 0.14%. Body mass index, waist-to-height ratio, blood pressure, triglycerides, LDL cholesterol, and hemoglobin A_1c_ were significantly higher in the highest quartile group of hemoglobin than in the lowest quartile group and tended to be higher with an increase of the quartile. Odds ratios of the highest versus lowest quartile groups of hemoglobin were 2.64 (2.25–3.10) for high LDL cholesterol/HDL cholesterol ratio, 3.05 (2.69–3.46) for high lipid accumulation product, 2.26 (2.05–2.50) for high cardiometabolic index, and 3.71 (3.07–4.47) for metabolic syndrome.

**Conclusions:**

Although the prevalence of polycythemia was very low, cardiometabolic risk was higher in those showing relatively high hemoglobin levels than in those with lower levels. Therefore, normal high blood hemoglobin is suggestive of increased cardiovascular risk in middle-aged women.

## Introduction 

Polycythemia, abnormal levels of hemoglobin and/or hematocrit, increases the risk of thrombotic cardiovascular events through hyper-viscosity of blood and increase in hemodynamic shear stress.^1,2^ There is a gender difference in the criteria of polycythemia vera: hemoglobin: >16.5 g/dL in men and >16.0 g/dL in women or hematocrit: >49% in men and >48% in women.^3^ A gender difference has also been shown in hemoglobin levels that were associated with an increased risk of major adverse cardiovascular events (men: ≥ 16.5 g/dL; women: ≥ 15.0 g/dL).^4^ On the other hand, U- or J-shaped associations between hemoglobin concentration and incidences of cardio- and cerebrovascular diseases were found in a prospective study using a large cohort of women.^5^ Moreover, lower hemoglobin levels were reported to be associated with a higher risk for adverse cardiovascular outcomes in women.^6^ Thus, there is a controversy in the findings of previous studies on the relationship between hemoglobin levels and the risk of cardiovascular diseases in women.

There has been an accumulation of information on the relationship of hemoglobin with cardiovascular risk factors. In a recent prospective study, hemoglobin was shown to be associated with metabolic syndrome, high triglycerides (TGs), low high-density lipoprotein cholesterol (HDL-C), and hyperuricemia but not with abdominal obesity, blood pressure, or fasting blood sugar in women.^7^ In a case–control study, higher hemoglobin was associated with metabolic syndrome and all of the components of metabolic syndrome in men and women.^8^ However, an association between hemoglobin and the incidence of metabolic syndrome has been found in men but not in women.^9^ In previous studies, positive associations between hemoglobin and blood pressure have been shown in men and women,^10–13^ while a recent prospective study in China has shown that a high level of hemoglobin was associated with an increased risk of hypertension in women but not in men.^14^ A cross-sectional study in Japan showed a positive association between hemoglobin and hypertension in nonanemic men and women.^15^ Thus, gender-related differences were also found in the relationships of hemoglobin with metabolic syndrome and hypertension. One possible reason for the above gender-related differences in the relationships of hemoglobin with cardiovascular diseases, metabolic syndrome, and hypertension is the inclusion of women with anemia in studies because their proportions and severities differed among the studies. However, little information has been reported on the relationship between blood hemoglobin levels and cardiovascular risk in women without anemia. Moreover, it remains unknown whether and how normal high levels of hemoglobin in blood are related to cardiometabolic risk in women.

There were two purposes of this study: One was to investigate the relationships of hemoglobin with cardiovascular risk factors including obesity indices, blood pressure, blood lipids, glycemic status, lipid-related indices, and metabolic syndrome in women without abnormally low hemoglobin levels (<11.0 g/dL) and the other purpose was to determine the cutoff value of hemoglobin to discriminate cardiometabolic risk.

## Methods

### Subjects

The overall participants were 18,410 Japanese female workers (35–60 years) who had undergone annual health checkups. The protocol of this cross-sectional study was approved by the Hyogo College of Medicine Ethics Committee (No. 3003).

Lifestyle factors including alcohol consumption, cigarette smoking, regular exercise (almost every day [30 minutes or longer per each day]), and histories of illnesses and medication therapies were surveyed by self-reported questionnaires for the participants. Individuals showing abnormally low hemoglobin levels (hemoglobin level of less than 11.0 g/dL) and/or having a history of therapy for anemia (*n* = 3,690) were excluded from the subjects. Finally, data for 14,720 participants were used for analysis.

The weekly average amount of alcohol intake was also reported in the questionnaire: Frequency of individual alcohol drinking was asked as “How frequently do you drink alcohol?” and was categorized as “every day” (regular drinkers), “sometimes” (occasional drinkers) and “never” (nondrinkers). Habitual smokers were defined as persons who had smoked cigarettes at least for the past month and for 6 months or longer. Smokers were categorized by daily average cigarette consumption as light smokers (20 cigarettes or less) and heavy smokers (21 cigarettes or more).

### Measurements of cardiometabolic risk factors

At the health checkup examination, height and body weight were measured. Body mass index (BMI) was calculated as weight in kilograms divided by the square of height in meters. Waist circumference was also measured at the navel level according to the definition of the Japanese Committee for the Diagnostic Criteria of Metabolic Syndrome,^16^ and visceral obesity was evaluated by using waist-to-height ratio defined as waist circumference (cm) divided by height (cm). Blood pressure was measured at rest in a sitting position with a mercury sphygmomanometer, and Korotkoff phase V was used for the definition of diastolic blood pressure. Fasted venous blood was collected from each participant, and serum was separated by centrifugation. A part of the whole blood was immediately transferred to a 2 mL glass tube containing 3.8 mg EDTA-2K. Hemoglobin concentration was measured by the sodium lauryl sulfate hemoglobin method using an automatic hematology analyzer (Sysmex XE-2100, Sysmex Corp., Kobe, Japan). Concentrations of TGs, HDL-C and low-density lipoprotein cholesterol (LDL-C) in the serum were measured using commercial kits, pureauto S TG–N, cholestest N-HDL, and cholestest LDL (Sekisui Medical Co., Ltd, Tokyo, Japan), respectively. Hemoglobin A_1C_ concentration was evaluated by the latex cohesion method and calibrated by using a formula proposed by the Japan Diabetes Society.^17^ Lipid accumulation product (LAP) was determined by using TG level and waist circumference (WC) as follows: LAP = TG (mmol/L) × [WC (cm) − 58].^18^ Cardiometabolic index (CMI) was defined as the product of two ratios: ratio of waist circumference to height (cm/cm) and ratio of TGs to HDL-C (mg/dL/mg/dL).^19^

### Criteria of cardiovascular risk factors

The definition of each risk factor was as follows: visceral obesity, waist-to-height ratio ≥0.5;^20^ hypertension, systolic blood pressure ≥140 mmHg and/or diastolic blood pressure ≥90 mmHg;^21^ abnormally low hemoglobin, hemoglobin <11.0 g/dL; polycythemia, hemoglobin >16.0 g/dL;^3^ high TGs, TGs ≥150 mg/dL; low HDL-C, HDL-C <50 mg/dL; high LDL-C, LDL-C ≥140 mg/dL;^22,23^ diabetes, hemoglobin A_1c_ ≥ 6.5%;^24^ and high CMI ≥0.800.^19^ Participants who were receiving medication therapy for hypertension and diabetes were also regarded as those with hypertension and diabetes, respectively. Metabolic syndrome was defined, according to the criteria of the International Diabetes Federation^25^ with a slight modification, as the presence of 2 or more risk factors in addition to visceral obesity diagnosed as high waist-to-height ratio. Risk factors included in the criteria are visceral obesity (high waist-to-height ratio), hypertension, dyslipidemia (low HDL-C and/or high TGs) and diabetes.

### Statistical analysis

The participants were divided into four almost equal-sized quartile groups after arranging them with hemoglobin levels in ascending order (1st quartile: 11.0–12.4 g/dL [*n* = 3,474]; 2nd quartile: 12.5–13.1 [*n* = 4,007]; 3rd quartile: 13.2–13.8 [*n* = 4,007]; and 4th quartile: 13.9–19.1 [*n* = 3,232]). Continuous variables are summarized as ranges, means with standard deviations or 95% confidence intervals, and medians with interquartile ranges, as appropriate. Categorical variables are summarized as frequencies and percentages. Cardiovascular risk-related variables were compared among the four quartile groups of hemoglobin by univariable and multivariable analyses as described below. In the univariable analyses, the continuous variables were compared among the four groups by using analysis of variance (ANOVA) followed by Scheffé’s F test as a *post-hoc* test. The categorical variables were compared by using Pearson’s chi-square test. In the multivariable analyses, the variables were compared among the four groups of hemoglobin by using analysis of covariance (ANCOVA) followed by Student’s *t-test* with Bonferroni’s multiplicity correction. Since TGs, LAP, and CMI did not display normal distributions, they were used for ANCOVA after being logarithmically transformed with a base of 10. The dichotomous variables were compared by using logistic regression analyses, in which the crude and adjusted odds ratios were estimated with their corresponding 95% confidence intervals. The multivariable analyses included adjustments for age, habits of smoking, alcohol drinking and regular exercise, history of medication therapy for hypertension, dyslipidemia or diabetes, and BMI as needed. Receiver operating characteristic (ROC) analysis was performed to examine an optimal cutoff point of hemoglobin for metabolic syndrome as an outcome. The area under the ROC curve (AUC) and 95% confidence interval were estimated empirically. The optimal cutoff point was selected by maximizing Youden’s index, which is the difference between the true positive rate (sensitivity) and the false positive rate (1-specificity) in the ROC curve. All probability (*p*) values were two-sided. Statistical significance was defined when a *p* value was less than 0.05. A computer software program (IBM SPSS Statistics for Windows, Version 25.0. Armonk, NY: IBM Corp) was used for the above-described statistical analyses.

## Results

### Characteristics of the subjects

**FIG. 1.**
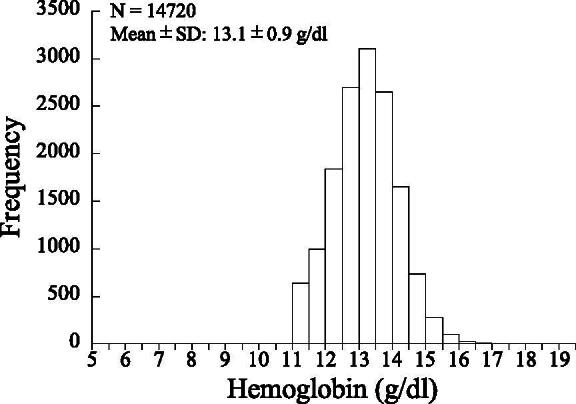
Histogram of hemoglobin concentrations in blood of subjects with analysis (*n* = 14,720).

Figure 1 shows a histogram of hemoglobin concentrations in the blood of overall participants. The percentage of participants showing polycythemia (>16 g/dL) was 0.14% (*n* = 25). Those showing abnormally low hemoglobin (<11 g/dL, *n* = 1,999 [10.9%]) and/or having a history of therapy for anemia (*n* = 2,485 [13.5%]) were excluded from the subjects. Table 1 shows the characteristics of the subjects with analysis. About 20% and 40% of the subjects were smokers and drinkers, respectively. The percentages of subjects with high BMI and high waist-to-height ratio were 21.1% and 47.5%, respectively. The percentages of subjects with hypertension, high LDL-C, diabetes, and metabolic syndrome were 21.0%, 20.0%, 2.8%, and 8.2%, respectively. The percentages of subjects receiving medication therapy for hypertension, dyslipidemia, and diabetes were 9.1%, 5.1%, and 1.5%, respectively.

**Table 1.** Characteristics of the Subjects

Variable	Number, means, medians, or percentages
Number	14720
Age (years)	49 (42, 54)
Smokers (%)	light, 18.2, heavy, 0.8
Alcohol drinkers (%)	occasional, 30.0; regular, 9.8
Regular exercise (%)	5.5
Therapy for hypertension (%)	9.1
Therapy for dyslipidemia (%)	5.1
Therapy for diabetes (%)	1.5
Height (cm)	156.2 ± 5.5
Weight (kg)	55.0 ± 9.4
BMI (kg/m^2^)	22.5 ± 3.7
High BMI (%)	21.1
Waist circumference (cm)	78.3 ± 9.8
Waist-to-height ratio	0.502 ± 0.065
High waist-to-height ratio (%)	47.5
Hemoglobin (g/dL)	13.1 ± 0.9
Systolic BP (mmHg)	122.2 ± 17.1
Diastolic BP (mmHg)	72.6 ± 11.3
Hypertension (%)	21.0
TGs (mg/dL)	80 (56, 118)
High TGs (%)	14.2
HDL-C (mg/dL)	65.7 ± 14.9
Low HDL-C (%)	13.0
LDL-C (mg/dL)	115.3 ± 30.5
High LDL-C (%)	20.0
LDL-C/HDL-C	1.87 ± 0.73
High LDL-C/HDL-C (%)	10.7
TG/HDL-C	1.22 (0.79, 2.02)
High TG/HDL-C (%)	20.6
LAP	17.0 (9.2, 31.4)
High LAP	19.2
CMI	0.60 (0.38, 1.05)
High CMI (%)	36.5
Hemoglobin A_1c_ (%)	5.38 ± 0.54
Diabetes (%)	2.8
Metabolic syndrome (%)	8.2

Shown are number, means with standard deviations, medians with interquartile ranges in parenthesis and percentages of the variables.

BMI, body mass index; BP, blood pressure; CMI, cardiometabolic index; HDL-C, high-density lipoprotein cholesterol; LAP, Lipid accumulation product; LDL-C, low-density lipoprotein cholesterol; TGs, triglycerides.

### Comparison of each variable related to cardiometabolic risk in the four quartile groups of hemoglobin

The percentages of light and heavy smokers, regular drinkers and subjects with a habit of regular exercise were significantly higher in the 3rd and 4th quartile groups of hemoglobin than in the lowest (1st) quartile group and tended to be higher with an increase of the quartile (Table 2). The percentages of subjects receiving therapy for hypertension, dyslipidemia, and diabetes were significantly higher in the 2nd, 3rd, and 4th quartile groups of hemoglobin than in the lowest (1st) quartile and tended to be higher with an increase of the quartile (Table 2). The percentages of subjects showing high BMI, high waist-to-height ratio, hypertension, high TGs, low HDL-C, high LDL-C, high LDL-C/HDL-C ratio, high TG/HDL-C ratio, high LAP, high CMI, diabetes, and metabolic syndrome were significantly higher in the 4th quartile group of hemoglobin than in the lowest (1st) quartile group and tended to be higher with an increase of the quartile (Table 3).

**Table 2.** Comparison of Percentages of Subjects with Each History Related to Cardiometabolic Risk in the Quartile Groups of Hemoglobin

	1st quartile	2nd quartile	3rd quartile	4th quartile
	(Hb: 11.0–12.4 g/dL)	(Hb: 12.5–13.1 g/dL)	(Hb: 13.2–13.8 g/dL)	(Hb: 13.9–19.1 g/dL)
Smokers (%)				
** **Light	14.2	15.3	18.4^**^	26.1^**^
** **Heavy	0.4	0.6	0.8^*^	1.7^**^
Alcohol drinkers (%)				
** **Occasional	28.6	30.1^*^	30.6^*^	30.9^**^
** **Regular	8.0	9.8^**^	9.9^**^	11.8^**^
Regular exercise (%)	4.2	5.4^*^	6.0^**^	6.1^**^
Therapy for hypertension (%)	5.4	8.0^**^	10.0^**^	13.6^**^
Therapy for dyslipidemia (%)	3.3	4.8^**^	5.6^**^	6.8^**^
Therapy for diabetes (%)	0.8	1.3^*^	1.4^*^	2.7^**^

^*^ *p* < 0.05.

^**^ *p* < 0.01.

Shown are percentages of each variable.

Asterisks denote significant differences from the 1st quartile group of hemoglobin.

Hb, hemoglobin.

**Table 3.** Comparison of Prevalences of Each Cardiometabolic Risk in the Quartile Groups of Hemoglobin

	1st quartile	2nd quartile	3rd quartile	4th quartile
	(Hb: 11.0–12.4 g/dL)	(Hb: 12.5–13.1 g/dL)	(Hb: 13.2–13.8 g/dL)	(Hb: 13.9–19.1 g/dL)
High BMI (%)	15.1	17.3^*^	21.6^**^	31.6^**^
High WHtR (%)	39.2	44.9^**^	49.1^**^	57.9^**^
Hypertension (%)	13.8	17.7^**^	22.1^**^	31.4^**^
High TGs (%)	9.0	12.5^**^	14.5^**^	21.6^**^
Low HDL-C (%)	11.5	11.7	12.9	16.2^**^
High LDL-C (%)	13.0	17.4^**^	21.3^**^	29.1^**^
High LDL-C/HDL-C (%)	7.0	9.1^**^	10.8^**^	16.5^**^
High TG/HDL-C (%)	14.4	19.1^**^	21.0^**^	28.7^**^
High LAP (%)	12.2	16.1^**^	19.8^**^	29.7^**^
High CMI (%)	28.5	33.7^**^	37.2^**^	47.4^**^
Diabetes (%)	1.6	2.0	2.6^**^	5.2^**^
MetS (%)	4.4	6.2^**^	8.3^**^	14.7^**^

^*^ *p* < 0.05.

^**^ *p* < 0.01.

Shown are prevalences of each cardiometabolic risk.

Asterisks denote significant differences from the 1st quartile group of hemoglobin.

CMI, cardiometabolic index; Hb, hemoglobin; HDL-C, high-density lipoprotein cholesterol; LAP, lipid accumulation product; LDL-C, low-density lipoprotein cholesterol; MetS, metabolic syndrome; TGs, triglycerides; WHtR, waist-to-height ratio.

### Comparison of mean levels of each cardiometabolic risk factor in the four quartile groups of hemoglobin

Mean levels of BMI, waist-to-height ratio, systolic and diastolic blood pressure, TGs, LDL-C, LDL-C/HDL-C ratio, TG/HDL-C ratio, LAP, and CMI were significantly higher in the 3rd and 4th quartile groups of hemoglobin than in the lowest quartile group and tended to be higher with an increase of the quartile (Table 4). The above results were not altered in multivariable analysis with adjustment for age, habits of smoking, alcohol drinking and regular exercise, and therapy for hypertension, dyslipidemia or diabetes (Table 4). Mean HDL-C and hemoglobin A_1C_ levels were slightly but significantly lower and higher, respectively, in the highest quartile of hemoglobin than in the lowest quartile in univariable analysis, but these differences were not found in multivariable analysis (Table 4).

**Table 4.** Comparison of Mean Levels of Each Variable in the Quartile Groups of Hemoglobin

	1st quartile	2nd quartile	3rd quartile	4th quartile
	(Hb: 11.0–12.4 g/dL)	(Hb: 12.5–13.1 g/dL)	(Hb: 13.2–13.8 g/dL)	(Hb: 13.9–19.1 g/dL)
BMI				
** **Univariable	21.9 (21.8–22.0)	22.2 (22.1–22.3)^*^	22.6 (22.5–22.7)^**^	23.6 (23.4–23.7)^**^
** **Multivariable	21.9 (21.8–22.1)	22.2 (22.1–22.3)^*^	22.6 (22.5–22.7)^**^	23.6 (23.5–23.7)^**^
WHtR				
** **Univariable	0.489 (0.487–0.491)	0.496 (0.494–0.498)^**^	0.503 (0.501–0.505)^**^	0.519 (0.517–0.522)^**^
** **Multivariable	0.491 (0.489–0.493)	0.496 (0.494–0.498)^**^	0.503 (0.501–0.505)^**^	0.519 (0.517–0.521)^**^
Systolic BP (mmHg)				
** **Univariable	118.9 (118.3–119.4)	120.1 (119.6–120.6)^*^	122.8 (122.3–123.3)^**^	127.5 (126.9–128.1)^**^
** **Multivariable	120.7 (120.2–121.2)	120.6 (120.1–121.1)	122.6 (122.1–123.0)^**^	125.2 (124.7–125.8)^**^
Diastolic BP (mmHg)				
** **Univariable	70.0 (69.6–70.4)	71.2 (70.9–71.5)^**^	73.1 (72.8–73.5)^**^	76.5 (76.1–76.9)^**^
** **Multivariable	71.0 (70.7–71.4)	71.5 (71.2–71.8)	73.0 (72.7–73.3)^**^	75.1 (74.8–75.5)^**^
Log(TG [mg/dL])				
** **Univariable	1.87 (1.86–1.87)	1.90 (1.89–1.91)^**^	1.93 (1.92–1.93)^**^	1.98 (1.97–1.99)^**^
** **Multivariable	1.89 (1.88–1.90)	1.91 (1.90–1.92)^**^	1.93 (1.92–1.93)^**^	1.95 (1.95–1.96)^**^
HDL -C (mg/dL)				
** **Univariable	65.9 (65.4–66.3)	66.1 (65.6–66.5)	65.9 (65.4–66.3)	64.8 (64.3–65.3)^*^
** **Multivariable	65.2 (64.8–65.7)	65.5 (65.1–65.9)	65.9 (65.4–66.3)	66.2 (65.7–66.7)^*^
LDL-C (mg/dL)				
** **Univariable	108.1 (107.2–109.0)	113.2 (112.3–114.1)^**^	117.1 (116.2–118.1)^**^	123.5 (122.4–124.6)^**^
** **Multivariable	110.0 (109.1–110.9)	113.7 (112.8–114.5)^**^	116.9 (116.1–117.8)^**^	121.2 (120.2–122.2)^**^
LDL-C/HDL-C				
** **Univariable	1.74 (1.72–1.76)	1.82 (1.80–1.84)^**^	1.89 (1.87–1.92)^**^	2.05 (2.02–2.07)^**^
** **Multivariable	1.79 (1.77–1.81)	1.85 (1.83–1.87)^**^	1.89 (1.87–1.91)^**^	1.96 (1.94–1.99)^**^
Log(TG/HDL-C)				
** **Univariable	0.059 (0.049–0.068)	0.092 (0.083–0.101)^**^	0.120 (0.111–0.129)^**^	0.184 (0.173–0.195)^**^
** **Multivariable	0.085 (0.076–0.094)	0.104 (0.095–0.112)^*^	0.118 (0.110–0.126)^**^	0.145 (0.135–0.154)^**^
Log(LAP)				
** **Univariable	1.131 (1.118–1.144)	1.188 (1.176–1.200)^**^	1.235 (1.223–1.247)^**^	1.343 (1.329–1.357)^**^
** **Multivariable	1.146 (1.134–1.159)	1.188 (1.176–1.200)^**^	1.231 (1.220–1.243)^**^	1.331 (1.318–1.344)^**^
Log(CMI)				
** **Univariable	−0.255 (−0.265 to −0.250)	−0.215 (−0.225 to −0.205)^**^	−0.182 (−0.191 to −0.172)^**^	−0.104 (−0.116 to −0.092)^**^
** **Multivariable	−0.220 (−0.229 to −0.210)	−0.200 (−0.209 to −0.192)^*^	−0.185 (−0.193 to −0.176)^**^	−0.157 (−0.166 to −0.147)^**^
Hemoglobin (%)				
** **Univariable	5.36 (5.35–5.37)	5.34 (5.33–5.36)	5.36 (5.35–5.38)	5.46 (5.43–5.49)^**^
** **Multivariable	5.40 (5.39–5.42)	5.36 (5.34–5.37)^**^	5.36 (5.35–5.37)^**^	5.40 (5.39–5.42)

^*^ *p* < 0.05.

^**^ *p* < 0.01.

Shown are means with 95% confidence intervals of each variable. In multivariable analysis, age and habits of smoking, alcohol drinking and regular exercise were used for covariates. BMI was adjusted in analyses of variables except for BMI, WHtR, log(LAP), and log(CMI). In addition, histories of medication therapy for hypertension, dyslipidemia and diabetes were adjusted in analyses of blood pressure, lipid-related variables and hemoglobin A_1c_, respectively.

Asterisks denote significant differences from the 1st quartile group of hemoglobin.

BMI, body mass index; CMI, cardiometabolic index; Hb, hemoglobin; WHtR, waist-to-height ratio; BP, blood pressure; TGs, triglycerides; HDL-C, high-density lipoprotein cholesterol; LAP, Lipid accumulation product; LDL-C, low-density lipoprotein cholesterol.

### Odds ratios for each cardiometabolic risk of the 2nd, 3rd, and 4th quartile groups versus the 1st quartile group of hemoglobin

Table 5 shows odds ratios of each quartile group versus the 1st quartile group of hemoglobin for variables related to cardiometabolic risk including high BMI, high waist-to-height ratio, hypertension, high TGs, low HDL-C, high LDL-C, high LDL-C/HDL-C ratio, high TG/HDL-C ratio, high LAP, high CMI, diabetes, and metabolic syndrome. Both in univariable and multivariable logistic regression analyses, the odds ratios of the highest (4th) versus lowest (1st) groups of hemoglobin for having the above variables of cardiometabolic risk factors except for low HDL-C were significantly higher than the reference level of 1.00 and tended to be higher with an increase of the quartile. The odd ratio of the highest versus lowest quartile groups of hemoglobin for low HDL-C was significantly higher than the reference level in univariable analysis but was not significantly different from the reference level in multivariable analysis.

**Table 5.** Odds Ratios for Each Cardiometabolic Risk of 2nd, 3rd, and 4th versus 1st Quartile Groups of Hemoglobin

	1st quartile	2nd quartile	3rd quartile	4th quartile
	(Hb: 11.0–12.4 g/dL)	(Hb: 12.5–13.1 g/dL)	(Hb: 13.2–13.8 g/dL)	(Hb: 13.9–19.1 g/dL)
High BMI				
Univariable	1.00	1.17 (1.03–1.32)^*^	1.55 (1.37–1.74)^**^	2.59 (2.30–2.92)^**^
Multivariable	1.00	1.16 (1.03–1.32)^*^	1.56 (1.38–1.76)^**^	2.71 (2.40–3.06)^**^
High WHtR				
Univariable	1.00	1.26 (1.15–1.39)^**^	1.50 (1.36–1.64)^**^	2.13 (1.93–2.35)^**^
Multivariable	1.00	1.19 (1.08–1.31)^**^	1.43 (1.30–1.57)^**^	2.06 (1.86–2.28)^**^
Hypertension				
Univariable	1.00	1.34 (1.18–1.52)^**^	1.76 (1.56–1.99)^**^	2.85 (2.52–3.22)^**^
Multivariable	1.00	1.12 (0.98–1.29)	1.40 (1.22–1.60)^**^	1.94 (1.69–2.22)^**^
High TGs				
Univariable	1.00	1.45 (1.25–1.68)^**^	1.73 (1.49–2.00)^**^	2.80 (2.43–3.23)^**^
Multivariable	1.00	1.28 (1.10–1.49)^**^	1.43 (1.23–1.66)^**^	1.95 (1.68–2.28)^**^
Low HDL-C				
Univariable	1.00	1.02 (.088–1.18)	1.14 (0.99–1.31)	1.50 (1.30–1.72)^**^
Multivariable	1.00	0.97 (0.84–1.12)	1.02 (0.88–1.18)	1.10 (0.94–1.28)
High LDL-C				
Univariable	1.00	1.42 (1.25–1.61)^**^	1.81 (1.60–2.05)^**^	2.76 (2.43–3.12)^**^
Multivariable	1.00	1.30 (1.14–1.49)^**^	1.58 (1.39–1.80)^**^	2.22 (1.94–2.54)^**^
High LDL-C/HDL-C				
Univariable	1.00	1.42 (1.25–1.61)^**^	1.62 (1.38–1.91)^**^	2.64 (2.25–3.10)^**^
Multivariable	1.00	1.21 (1.01–1.45)^*^	1.35 (1.13–1.60)^**^	1.83 (1.54–2.18)^**^
High TG/HDL-C				
Univariable	1.00	1.40 (1.24–1.59)^**^	1.59 (1.40–1.79)^**^	2.39 (2.12–2.70)^**^
Multivariable	1.00	1.28 (1.12–1.46)^**^	1.34 (1.18–1.52)^**^	1.65 (1.45–1.89)^**^
High LAP				
Univariable	1.00	1.39 (1.22–1.59)^**^	1.78 (1.56–2.02)^**^	3.05 (2.69–3.46)^**^
Multivariable	1.00	1.30 (1.14–1.49)^**^	1.66 (1.46–1.89)^**^	2.81 (2.47–3.21)^**^
High CMI				
Univariable	1.00	1.28 (1.16–1.41)^**^	1.49 (1.35–1.64)^**^	2.26 (2.05–2.50)^**^
Multivariable	1.00	1.20 (1.08–1.33)^**^	1.40 (1.27–1.55)^**^	2.07 (1.86–2.30)^**^
Diabetes				
Univariable	1.00	1.27 (0.90–1.79)	1.66 (1.19–2.30)^**^	3.43 (2.52–4.67)^**^
Multivariable	1.00	1.05 (0.73–1.50)	1.17 (0.83–1.65)	2.13 (1.53–2.94)^**^
MetS				
Univariable	1.00	1.42 (1.15–1.74)^**^	1.95 (1.61–2.38)^**^	3.71 (3.07–4.47)^**^
Multivariable	1.00	1.27 (1.03–1.57)^*^	1.73 (1.42–2.12)^**^	3.42 (2.82–4.15)^**^

^*^ *p* < 0.05.

^**^ *p* < 0.01.

Shown are odds ratios with 95% confidence intervals in parentheses. In multivariable analysis, age and habits of smoking, alcohol drinking and regular exercise were used for explanatory variables. BMI was adjusted in analyses of variables except for high BMI, high WHtR, high LAP, and high CMI. In addition, a history of medication therapy for dyslipidemia was adjusted in analyses of lipid-related variables except for metabolic syndrome.

Asterisks denote significant differences from the reference level of 1.00.

BMI, body mass index; CMI, cardiometabolic index; Hb, hemoglobin; HDL-C, high-density lipoprotein cholesterol; LAP, Lipid accumulation product; LDL-C, low-density lipoprotein cholesterol; MetS, metabolic syndrome; TGs, triglycerides; WHtR, waist-to-height ratio.

### ROC analysis for the relationship between hemoglobin and metabolic syndrome

**FIG. 2.**
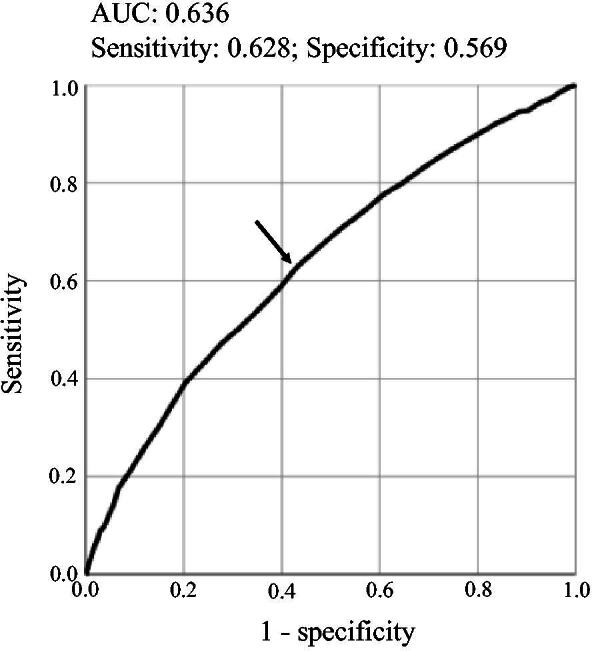
Receiver operating characteristic (ROC) curve for the relationship between hemoglobin levels and metabolic syndrome in middle-aged women without abnormally low hemoglobin levels. The arrow in the figure indicates the cutoff point. Area under the ROC curve (AUC) and sensitivity and specificity of the cutoff point are shown in the figure.

ROC analysis was performed to determine the cutoff value of hemoglobin using metabolic syndrome as an outcome (Fig. 2). The AUC value was 0.636 (95% confidence interval: 0.619–0.652) and the cutoff value of hemoglobin was 13.25 g/dL with sensitivity and specificity of 0.628 and 0.569, respectively.

## Discussion

In middle-aged nonanemic women, high hemoglobin levels were associated with known cardiovascular risk factors, including obesity, hypertension, dyslipidemia and diabetes, and were associated with indices of cardiometabolic risk including LDL-C/HDL-C ratio, TG/HDL-C ratio, LAP, and CMI as well as metabolic syndrome. The results of multivariable analyses indicated that these associations between hemoglobin levels and cardiometabolic risk factors were independent of possible confounders, age, lifestyles including smoking, alcohol drinking and regular exercise, and medication therapy for hypertension, dyslipidemia, and diabetes. Therefore, normal high hemoglobin is thought to be a cardiovascular risk factor in women, although the prevalence of polycythemia is very low (0.14% in the present study).

Among the above cardiovascular risk factors, only low HDL-C did not show a significant association with high hemoglobin in multivariable analyses. We also performed logistic regression analysis for the relationship between hemoglobin and low HDL-C using its less strict criterion of <40 mg/dL, which is used for both men and women in the annual lifestyle health checkups for the prevention of cardiovascular diseases managed by the government in Japan. The odds ratios with their 95% confidence intervals for low HDL-C in the 2nd, 3rd, and 4th quartiles versus the 1st quartile of hemoglobin were 0.87 (0.61–1.22), 0.81 (0.57–1.14), and 0.86 (0.60–1.21), respectively, in multivariable analysis adjusting for age, BMI, therapy for dyslipidemia, and habits of smoking, alcohol drinking and regular exercise. Thus, this finding of no significant relationship between high hemoglobin and low HDL-C was similar to the result in the analysis using the criterion of low HDL-C of <50 mg/dL (Table 5) and agrees with the finding of our previous study that the odds ratio for low HDL-C of subjects with versus those without polycythemia was not significantly different from the reference level in middle-aged men.^26^ Thus, both in women and men, low HDL-C, a cardiovascular risk factor, is not associated with high hemoglobin. On the other hand, hemoglobin levels showed an association with levels of TGs, and TG levels are known to be associated with HDL-C levels.^27^ Since HDL-C level is known to be affected potently by smoking and alcohol drinking,^28^ the relationships between hemoglobin and low HDL-C was investigated in subjects without habits of smoking and alcohol drinking. The odds ratio for low HDL-C of the 4th versus 1st quartiles of hemoglobin was not significantly different from the reference level (odds ratio: 0.91 [0.74–1.12]). Thus, hemoglobin was not associated with low HDL-C, which was independent of smoking and drinking, and the reason for no association between hemoglobin and HDL-C remains unknown.

Since obesity is the central risk factor of cardiometabolic disorders and an essential factor of metabolic syndrome,^16^ there was a possibility that obesity confounded the relationships between other cardiometabolic risk factors and hemoglobin. However, this is unlikely because the above relationships remained significant in multivariable analysis with adjustment for BMI. Smoking is known to be associated with polycythemia^29^ and dyslipidemia^30^ and was therefore a possible confounding factor for the relationships between hemoglobin and blood lipids including TGs and LDL-C. However, this possibility is also unlikely according to the results of multivariable analysis with adjustment for smoking (Tables 4 and 5). In logistic regression analysis, relatively high odds ratios of the 4th versus 1st quartile groups of hemoglobin were obtained for high LAP (3.05 [2.69–3.46]) and metabolic syndrome (3.71 [3.07–4.47]), which is reasonable because plural risk factors composing LAP and metabolic syndrome were associated with hemoglobin levels.

In the cohort used in this study, there were very few participants with polycythemia (0.14%). Thus, the hemoglobin levels of most of the subjects were not abnormally high (16.0 g/dL or lower). However, levels of cardiometabolic risk factors tended to increase with an increase of the quartile for hemoglobin. Therefore, normal high hemoglobin is thought to be a risk factor for cardiovascular diseases in middle-aged women. We tried to determine the cutoff value of hemoglobin for discrimination of metabolic syndrome by ROC analysis. Although the cutoff value was calculated to be 13.25 g/dL, the AUC was not large enough (0.636) and good accuracy of AUC was not obtained (sensitivity, 0.628; specificity, 0.569). Further studies using other outcomes, instead of metabolic syndrome, in ROC analysis are needed to determine more accurate cutoff values of hemoglobin for prevention of cardiovascular diseases in women.

There are limitations of this study. The number of subjects showing polycythemia was too small to investigate the relationships between polycythemia and cardiometabolic risk in this study. The cutoff value for abnormally low hemoglobin used in this study was 11.0 g/dL, which is lower than the cutoff value for anemia of adult women (12.0 g/dL) by the WHO criterion.^31^ Thus, there was a relatively large number (*n* = 2,042, 11.1%) of subjects who showed borderline levels (11.0–12.0 g/dL) of hemoglobin concentrations in this study. The subjects were Japanese middle-aged (35–60 years) women, and further studies using databases of subjects at younger and older ages and with other ethnicities are needed to confirm the findings of the present study. Menopause has been shown to be a crucial factor related to changes in concentrations of blood lipids and hemoglobin in a recent longitudinal study.^32^ Therefore, another limitation of the present study is that there was no categorization of the subjects for pre-, peri-, or postmenopausal. Information on individual menstrual status, which influences hemoglobin level, was not available in this study. In the multivariable analysis, lifestyle factors including smoking, alcohol drinking and regular exercise as well as age, obesity and medication therapy were adjusted; however, there are other possible confounders, *e.g.,* nutrition and socioeconomic factors, for which information was not available in this study. Finally, this study is cross-sectional in its design and further prospective studies are needed to discuss causal relationships between hemoglobin levels and cardiometabolic risk.

In conclusion, the prevalence of polycythemia (hemoglobin concentrations: >16.0 g/dL) was very low (0.14%) in middle-aged women. However, cardiometabolic risk was higher in individuals with relatively high hemoglobin levels than in those with lower hemoglobin levels. The associations of a high level of hemoglobin with hypertension, dyslipidemia (high TGs and high LDL-C), diabetes and metabolic syndrome were independent of smoking and obesity. Thus, normal high hemoglobin in peripheral blood is thought to be a cardiometabolic risk factor in middle-aged women.

## Data Sharing Statement

Deidentified individual subject data will not be made available.

## Abbreviations Used

ANCOVA: analysis of covariance
ANOVA: analysis of variance
AUC: area under the curve
BMI: body mass index
BP: blood pressure
CMI: cardiometabolic index
Hb: hemoglobin
HDL-C: high-density lipoprotein cholesterol
LAP: lipid accumulation product
LDL-C: low-density lipoprotein cholesterol
MetS: metabolic syndrome
ROC: receiver operating characteristic
TGs: TGs
WC: waist circumference
WHtR: waist-to-height ratio
